# Contextual explanations for numeracy and literacy skill disparities between native and foreign-born adults in western countries

**DOI:** 10.1371/journal.pone.0172087

**Published:** 2017-03-16

**Authors:** Mark Levels, Jaap Dronkers, Christopher Jencks

**Affiliations:** 1 Maastricht University, Maastricht, The Netherlands; 2 College for Interdisciplinary Education Research, Berlin, Germany; 3 Nuffield College, Oxford, United Kingdom; 4 Kennedy School of Government, Harvard University, Cambridge (MA), United States of America; Tilburg University, NETHERLANDS

## Abstract

Using new direct measures of numeracy and literacy skills among 85,875 adults in 17 Western countries, we find that foreign-born adults have lower mean skills than native-born adults of the same age (16 to 64) in all of the examined countries. The gaps are small, and vary substantially between countries. Multilevel models reveal that immigrant populations’ demographic and socioeconomic characteristics, employment, and language proficiency explain about half of the cross-national variance of numeracy and literacy skills gaps. Differences in origin countries’ average education level also account for variation in the size of the immigrant-native skills gap. The more protective labor markets in immigrant-receiving countries are, the less well immigrants are skilled in numeracy and literacy compared to natives. For those who migrate before their teens (the 1.5 generation), access to an education system that accommodates migrants’ special needs is crucial. The 1 and 1.5 generation have smaller numeracy and literacy skills gaps in more ethnically diverse societies.

## 1. Introduction

This paper explores disparities in numeracy and literacy skills between adult immigrants and natives in 17 Western countries. These skills are considered increasingly crucial for successful participation in contemporary Western societies and labor markets [1]. Numeracy and literacy are important predictors of individuals’ educational and occupational attainment, their economic productivity [2], their health [3], and their social participation [4]. At the macro level, ethnic and racial disparities in general skills explain a wide variety of intergroup inequalities. For example, ethnic and racial disparities in reading and math at school entry predict disparities in eventual educational attainment quite accurately (see for example: [5]. Similarly, ethnic and racial wage disparities in the US and Canada can also be largely attributed to differences in observed skills ([6,7].

Immigrants are on average less proficient in literacy and numeracy skills than natives, which goes a long way in explaining why immigrants often face problems integrating economically and socially into their host countries [8,9]. This robust empirical regularity has attracted much scholarly attention. Most studies aimed at explaining skills gaps between migrants and non-migrants rely on data about *immigrant children and the children of immigrants* [10–16]. Such studies are particularly revealing about the essential role formal education in receiving countries plays in equipping second generation immigrants with the skills necessary for future participation in the societies to which their parents have moved [17].

Nonetheless, studies on second generation immigrant children can tell only part of the story of numeracy and literacy skills disparities between immigrants and natives. For a number of practical and theoretical reasons, gaining more knowledge about adult first generation immigrants’ relative performance is also important. First, first generation immigrants (people who are themselves born in other countries than the country they reside in and who have immigrated to a new country of residence) have very different integration experiences in destination countries’ societies. The children of immigrants can use formal education as an important pathway for integrating into host societies [17–19]. Although second generation immigrants (immigrants who are born in their destination countries but whose parents are born abroad) are still partly socialized in origin country culture through their parents, school is an environment where they can absorb their destination countries’ culture and build social capital. First generation immigrants who arrive in their destination country as adults often do not have access to the education system. Instead, they have to integrate directly into the labor market and society of the receiving country. Studies of second generation immigrant children do not inform us about the role that labor market arrangements, integration policies, and social forces play in helping immigrants to close skills gaps in destination countries.

Secondly, it is important to learn more about the role of education in perpetuating or closing numeracy and literacy skills gaps between native-born children and foreign-born immigrants who migrate as a child or in their teens. Usually these so-called 1.5 generation immigrants [20] have access to education in host countries, but the role education plays in their integration is likely to differ from its role for the children born to immigrants after they arrive in the receiving country. Because first generation immigrant children have spent some part of their lives in their country of origin, because they have actually undergone the usually stressful experience of moving to a new country, because their parents have much less knowledge about the education system in their destination country, and because their parents partially socialize them into the culture in which they themselves were raised, schooling affects cognitive skills of immigrant children differently [21]. Existing studies on numeracy skills of first generation immigrant children (see e.g. [22]) compare their skills with those of immigrant children of the second generation, but they do not directly compare these children’s skills to those of natives.

To understand skill gaps between first generation adult immigrants and natives, it helps to consider two main drivers for these gaps. First, immigrants have different starting points than natives. Second, after arrival immigrants may follow different trajectories than natives [23]. Both starting points and trajectories may depend on macro-level characteristics of sending and receiving countries. The fact that such country-level contextual differences affect immigrants’ starting points is well established. For example, the size of the initial skills gap depends partly on the receiving country’s immigration policies. Literacy skills that immigrants obtained in their origin country and other context-specific skills and information may not be readily transferable to their destination country [24–27]. First generation immigrants who are more proficient in their native language may, for example, be less proficient than natives in reading the language of their new country, and this is likely to affect their performance on math tests. In countries with selective immigration policies, immigrants’ human capital is usually of higher quality, reducing the initial numeracy and literacy skill gaps [22,28]. A variety of theories also predict that other contextual differences will affect immigrant trajectories. For example, integration policies that aim at stimulating the social and cultural integration of immigrants and reducing human capital disparities over time vary from one host country to another. Cultural and ethnic diversity may provide opportunities for contact, but also for avoiding contact. Different education systems and labor markets thus may provide immigrants with quite different trajectories into society.

Quantitative analyses of contextual explanations for disparities in numeracy and literacy skills between adult first generation immigrants and natives are scarce and usually on a single receiving country, such as Canada [7], the Netherlands [29], or the US [30]. This limits their use for testing hypotheses about destination country differences. Available cross-national studies compare only a limited number of countries, and disregard origin differences in skills. Because it is hard to model cross-level interactions between context and migrant status, studies that compare only a handful of countries must be treated cautiously.

In this paper, we answer three research questions:

1. *To what extent is there a gap in numeracy and literacy skills between adult natives and either first generation or 1*.*5 generation immigrants and natives*,
2. *To what extent does the size of these gaps vary across affluent Western nations*?
3. *To what extent can cross-national variation in numeracy and literacy skill gaps be predicted by contextual characteristics of receiving countries*?

To answer these questions, we analyze data from OECD’s Programme for the International Assessment of Adult Competencies (PIAAC) [31]. PIAAC is a large cross-national survey of adult skills, conducted among adults aged 16 to 65 in 24 countries. It measures demographic and socio-economic characteristics of individuals, and provides direct measures of cognitive skills. PIAAC is also the first data set with sufficiently large samples of first generation adult immigrants in enough countries to allow robust tests of complex explanations for the skills gaps between natives and first-generation adult immigrants in different nations. We use these data to describe the size of the numeracy and literacy achievement gap between natives, first generation immigrants who migrated as adults, and first generation immigrants who migrated as children (1.5 generation) in 17 Western countries.

We then combine the PIAAC data with high-quality macro-level indicators of countries’ relevant policies and institutional characteristics and analyze these pooled data using multivariate (hierarchical) multilevel models [32]. The purpose of the analyses is not to predict immigrant literacy or numeracy skills, but to analyze how their skills compare to natives in various countries. This allows us to we test a variety of social-scientific theories explaining skills disparities between natives and immigrants, including assimilation theory [33], theories on the relevance of origin country differences, ethnic contact theory [34,35], constrict theory [36], and institutional theories that point toward the relevance of educational and labor market institutions.

We contribute to the literature in three further ways. First, our study is relevant for research in economics and sociology on human capital differences between first generation immigrants and natives. Most of those studies rely on indirect measures of productivity-enhancing skills, such as occupational attainment [37], educational attainment [38] or wages [39]. Such proxies are not ideal for measuring human capital in cross-national settings. The quality of education within the same level of schooling and the selectivity for attending a given level of school differ markedly between among both sending and receiving countries [40], so that the average increase in skills associated with each year of additional schooling differs across countries. Wages and occupational status are also unavailable for people without jobs, and may be biased due to origin-related discrimination [41]. By using direct and cross-nationally comparable measures of numeracy and literacy skills, our design avoids these problems. The PIAAC tests are taken in the national languages of the test country, and as such measures the proficiency of the adult population in reading and in working with numbers in the language(s) that are most relevant to participation in the economic and civic life of the country in which immigrants live [42].

Second, compositional differences between immigrant and native subpopulations might explain large portions of the cross-national variation in skills gaps between immigrants and natives ([11,22,39,43] Compositional differences emerge if natives and first generation immigrants differ with regard to other individual-level characteristics associated with numeracy skills. These individual differences aggregate into inter-group differences. Research shows that aggregate differences related to demographic makeup and health, the level of educational attainment, socioeconomic class, employment, ethnicity, and migration differences explain a large part of the skill gaps between natives and immigrants, both in the US [23] and other OECD countries [14,22,44]. Fryer and Levitt [45] show that family background characteristics almost fully explain the initial gap in test scores between black children and white children in the US. By adopting a multilevel perspective, we can determine the extent to which observed cross-national variation in numeracy and literacy skills disparities are explained by compositional differences of populations, and how the remaining variation is associated with contextual differences. A multi-level perspective reduces bias in measuring the sizes of parameters and increases the power of our tests for both contextual and compositional explanations.

Third, we take the role of origin country differences into account. Cultural differences in migrants’ origin are important factors for explaining immigrant integration into host societies [46]. Origin differences also appear crucial for explaining math skill disparities between natives and immigrant children [13]. US research suggests that earnings disparities between adult immigrants and natives can partly be attributed to origin group differences [47,48]. Our analyses explore the relevance of origin countries’ average educational attainment in predicting cross-national variation in numeracy and literacy skill gaps between natives and first generation immigrants.

## 2. Theoretical explanations and hypotheses

To formulate hypotheses we draw from assimilation theory [33], ethnic contact theory [34,35], constrict theory [36], and various institutional theories. These theoretical explanations are useful for explaining a wide variety of empirical phenomena, but have in common that they all presuppose the relevance of receiving countries’ characteristics for immigrant integration.

We distinguish two types of country differences. First, explanations for literacy and numeracy skill gaps may pertain to differences in individuals’ characteristics that aggregate into compositional differences between groups of immigrants and natives. If natives and immigrants differ on average with regard to individual-level characteristics associated with learning abilities, these differences are likely to explain some of the observed numeracy and literacy skills disparities between the groups in various countries. Second, explanations may pertain to country-level characteristics that cannot be disaggregated into individual-level characteristics. Examples of such contextual effects include policies, laws, and institutional characteristics. Such macro explanations can relate to contextual characteristics of receiving countries, origin countries, and various combinations thereof (*cf*. [49]).

In this section we deduce hypotheses about the role receiving countries’ social and cultural characteristics and the structure of their labor markets and educational systems have in explaining cross-national variation of and literacy numeracy skills disparities between first and 1.5 generation immigrants and natives. We also formulate hypotheses about the average education level of origin countries’ populations. All hypotheses assume that compositional and contextual characteristics of origin and destination countries may influence both the size of numeracy gaps when immigrants arrive, but also that they may predict differences in what immigrants and natives learn in the receiving country [23].

### Compositional differences

Economic theories of first generation immigrants’ labor market success highlight that the skills immigrants have acquired in their origin country may not be fully transferable to their destination country [24–27]. Particularly language skills translate poorly: people who are highly proficient in their native language may be poorly proficient in the language of countries to which they migrate. This may negatively affect the way in which they can make use of other skills, such as numeracy of problem solving (see for example [42], p.76)

This implies that first generation immigrants and natives of the same age may have different starting -values of country specific numeracy and literacy skills [23]. Selective migration predicts that the size of the initial gap varies between countries. Classic assimilation theory predicts that over time different ethnic groups are all drawn into their host societies, and begin to share a common culture [50,51]. Later scholars extended the idea of assimilation, and predicted that over their life-course and with each successive generation, immigrants would also become more like natives on social-structural and economic dimensions [33,52]. A number of individual characteristics associated with these dimensions are strongly associated with skills: people with a higher education level, higher socio-economic background, better health, better language proficiency, and active employment status on average have better cognitive skills [1]. It follows that in countries where the immigrant subpopulation is more similar to natives on these characteristics, cognitive skills disparities should be smaller.

**Hypothesis 1:** numeracy (1a) and literacy (1b) skills disparities between natives and first and 1.5 generation adult immigrants are smaller in countries in which immigrants on average more closely resemble natives socioeconomically and demographically.

### Origin countries

The relation between origin country characteristics and skill gaps between immigrants and natives in destination countries has long been understudied [43, 53]. US research suggests that characteristics of the countries of origin, such as economic level, political stability, income distribution, literacy rate, and speaking English all affect the size of earnings differences between natives and immigrants [47,48], as well as educational attainment differences between immigrant and native children [53]. These country of origin differences are important over and above immigrants’ own socioeconomic backgrounds [47,49]. Origin country effects are persistent: cross-national research also highlights that origin differences partly explain mathematical literacy differences between immigrant children from different countries of origin in different destination countries [22]. The extent to which origin country differences also predict skills disparities between adult first generation immigrants and natives remains to be established. We focus here on the average level of education in people’s countries of birth.

**Hypothesis 2:** numeracy (2a) and literacy (2b) skills disparities between natives and first and 1.5 generation adult immigrants are smaller in destination countries where immigrants more often come from countries in which the general education level is closer to the general education level of the destination country.

### Integration policies

Prejudice against minorities varies across destination countries. Western countries legally prohibit ethnic and racial discrimination, but enforcing such laws is often both difficult and uneven. In addition, subtler forms of discrimination that are hard to regulate legally can still affect immigrants’ chances of social and economic integration [35,54]. The perception of cultural differences in others evokes prejudice [55], which hampers inter-ethnic contact [56]. Although immigrant groups may respond to perceived discrimination by attempting to outperform natives, discrimination can also undermine immigrants’ motivation, participation, and achievement [57].To stimulate immigrant integration, many countries have adopted laws and policies designed to counter subtle discrimination. If such policies and laws are effective, first generation immigrants should integrate more easily into societies and labor markets that have such rules (unless such policies are adopted more often by countries where integration has proven unusually difficult). Evidence on the relation between anti-discrimination laws on immigrants integration is inconclusive [58,59]. We will explore whether such laws and policies predict the size of cognitive skills gaps between native born and foreign-born adults.

**Hypothesis 3:** numeracy (3a) and literacy (3b) skills disparities between natives and first and 1.5 generation adult immigrants are smaller in countries that more strongly have adopted laws and policies to stimulate integration of immigrants.

### Education system

Immigrant children from the 1.5 generation have very specific educational needs [21, 60].They are generally less proficient than natives in the language(s) of their destination countries, less familiar with destination countries’ school cultures, and more often find themselves in schools that are not conducive to learning among either immigrants or natives. They also have to overcome the social and psychological burdens that generally come with adapting to new societies. Nonetheless, many studies have found that ceteris paribus, immigrant children perform surprisingly well compared to native children [13, 61]. Large differences between origin groups exist [13,43], but better-than-expected average performance may be explained by positive self-selection among migrant parents, by the high educational aspirations they have for their children, by their strong work-ethic, and by their relatively stable families [21]. Where immigrants do not succeed in education, this is often because they have less access to information, resources and opportunities [62]. Some countries’ educational systems are better suited than others for dealing with the specific circumstances immigrant children face [15,21].Portes and Fernández-Kelley [63], for example, hypothesize that an education system that supports teachers and counsellors who take an interest in disadvantaged immigrant children may give these children a better starting position. Similarly, a system in which educational tracking on ability is implemented only in a limited way may benefit immigrant children. Characteristics of secondary education systems predict a notable part of observed skills disparities between natives and immigrant children aged 15 [15]. US research strongly suggests that these different early-life starting positions have long-term consequences and contribute to explaining native-immigrant disparities in educational attainment and labor market outcomes [23]. This indicates that initial differences associated with education system traits may be persistent over time, and may also contribute to explaining skills gaps between adult 1.5 generation immigrants and natives.

**Hypothesis 4:** numeracy (4a) and literacy (4b) skills disparities between natives and 1.5 generation adult immigrants are smaller in countries in which the educational system is more capable to meet educational demands of immigrant children.

### Labor market protectionism

The way labor markets are structured is also a major source of differences between affluent Western societies. First-generation migrants have strong incentives to invest in gaining skills that are useful in their destination countries, perhaps particularly if they have to compensate for the less-than-perfect transferability of the skills they learned in their origin country. For first generation adult immigrants, most informal learning of new skills takes place in the labor market, making labor market participation of immigrants crucial for reducing skills disparities between immigrants and natives. Countries differ in the extent to which they allow immigrants to work, and in the extent to which they grant working immigrants the same rights as working natives. Furthermore, labor market protection often benefits those who already have jobs over those who are trying to get jobs, because employers say they are less willing to hire someone when the law makes it more burdensome to fire them if they do not meet the requirements of the job. As a result, first generation immigrants, who are almost by definition outsiders in their destination countries, have more difficulties gaining access to labor markets if existing workers are more protected [64–66]. This would reduce their opportunities for informal learning,

**Hypothesis 5**: numeracy (5a) and literacy (5b) skills disparities between natives and first and 1.5 generation adult immigrants are larger in countries with stronger labor market protection of workers.

### Ethnic diversity

The desire to participate in social life, make friends, and build social capital may provide immigrants with a strong incentive to invest in acquiring skills that enable one to interact with others This mechanism predicts that skills gaps between natives and immigrants can be associated with countries’ ethnic diversity in two theoretically distinct ways. First, according to *constrict theory* [36], ethnic diversity in communities causes people to avoid social contacts. Because people are generally drawn to people who resemble themselves, more ethnically homogeneous communities would more strongly facilitate social interactions [67]. Ethnic diversity is often thought to erode social capital and trust and cause people to hunker down [36,68,69] Such withdrawal reduces opportunities for interethnic contacts and may hinder integration between natives and immigrants, as it reduces incentives for immigrants to invest in acquiring skills, as well as reduces opportunities for informal learning of language skills. This in turn could prevent cognitive skills gaps from narrowing.

**Hypothesis 6**: numeracy (6a) and literacy (6b) skills disparities between natives and first and 1.5 generation adult immigrants are larger in countries with a greater ethnic diversity.

By contrast, *ethnic contact theory* [34,70] would predict that higher ethnic diversity is associated with a higher likelihood that people have social contacts with people from different ethnic backgrounds. A considerable body of empirical research supports this theory [35]. The higher ethnic diversity is, the more opportunity people have for interacting with people from other ethnicities, and the more probable interethnic contacts and even friendships become [71]. If social interaction between natives and immigrants is more likely in more highly diverse societies, first generation immigrants should face less trouble integrating.

**Hypothesis 7:** numeracy (7a) and literacy (7b) skills disparities between natives and first and 1.5 generation adult immigrants are smaller in countries with a higher ethnic diversity.

### Cultural diversity

Many Western countries (e.g. Belgium, Canada, the U.S., and Switzerland) traditionally had multicultural populations, with a number of different cultural groups living within their borders for generations. The “age of migration” [72] has further increased the cultural diversity of these and almost all other Western countries [66]. Various sociological and economic theories predict that cultural diversity hampers the social and economic integration of first generation immigrants into their destination countries. According to classical assimilation theory, cultural assimilation is necessary for socioeconomic integration [52], which implies that skill gaps should be larger in more culturally diverse countries. Indeed, countries that take a multiculturalist stance toward immigrants–by embracing cultural diversity and equally recognizing different cultural groups–appear to reduce incentives to invest in learning destination countries’ languages and engage in interethnic contacts, particularly in combination with strong welfare states [73,74].

**Hypothesis 8**: numeracy (8a) and literacy (8b) skills disparities between natives and first and 1.5 generation adult immigrants are larger in countries with a higher cultural diversity.

## 3. Data

Testing our hypotheses requires a large dataset with a large number of destination countries, enough adult respondents from both immigrant and non-immigrant backgrounds, and direct measures of their numeracy and literacy skills. The 2013 wave of the Programme for the International Assessment of Adult Competencies [PIAAC] survey is the first dataset that meets these requirements. The OECD collected these data in 2012 and 2013 from over 150,000 respondents in 24 highly industrialized countries [75].

Representative national samples contain over 5,000 adults between the age of 16 and 65, along with highly detailed information about a wide variety of background variables. The PIAAC uses advanced psychometric tests to provide reliable estimates of adults’ proficiency in literacy, numeracy and problem-solving in technology-rich environments. Respondents were asked to complete assessment tests designed to measure cognitive skills in these three domains. All three types of skills are essential for processing information [1].

Participation in PIAAC is voluntary, information is fully anonymized, and all respondents have agreed that their information may be used for scientific research without their further consent. For this reason, we did not seek testing by an ethical board before undertaking this research. While most PIAAC countries have a sizable proportion of immigrants in their samples, we had to exclude data from Poland, Slovakia, Japan and Korea, because their samples did not include enough immigrants to allow for reliable comparisons. We excluded Australia because the Australian PIAAC data come with a large set of additional legal stipulations that are incompatible with our analyses. Finally, the original Canadian sample included some 25,000 cases, which is about five times larger than samples from the other countries. To make the national sample more comparable in size to the national samples of the other countries, we drew a random 20% subsample from the original Canadian sample. Finally, we deleted individuals (N = 1,189) with missing values on birth countries. The total working sample contains 85,875 respondents (first and 1.5 generation immigrants and natives) from 17 countries with comparable sample sizes: Austria, Belgium, Canada, Czech Republic, Denmark, Estonia, Finland, France, Germany, Ireland, Italy, the Netherlands, Norway, Spain, Sweden, the United Kingdom, and the United States.

## 4. Measurements

This section will discuss the variables we use. Descriptive statistics appear in Table 1.

**Table 1:** Descriptive statistics.

Individuals	N	Minimum	Maximum	Mean	Std. Deviation
1 generation immigrant	85875	0	1	0.09	0.29
1.5 generation immigrant	85875	0	1	0.02	0.15
One native parent	85875	0	1	0.01	0.10
Male	85875	0	1	0.48	0.50
Age	85875	16	65	41.13	14.18
Married	85875	0	1	0.61	0.49
Poor health	85875	0	1	0.04	0.19
Educational attainment	85875	0	19	12.72	3.11
Parents middle education	85875	0	1	0.35	0.48
Parents higher education	85875	0	1	0.24	0.43
Parents education unknown	85875	0	1	0.06	0.23
Unemployed	85875	0	1	0.06	0.23
Work experience	85875	0	55	18.24	13.58
Foreign language spoken at home	85871	0	1	0.09	0.28
**Origin countries**					
General education level	85053^a^	0.05	13.51	9.33	2.21
**Destination countries**					
Average numeracy score	85875	243	285	268.38	12.71
Integration policies	85875	41	83	58.58	10.31
Education system accommodation of immigrants	79970^b^	1	43	14.00	10.67
Labor market protectionism	85875	0.85	3.11	2.14	0.67
Ethnic diversity	85875	0.06	0.71	0.24	0.20
Cultural diversity	82174^c^	0.04	0.5	0.21	0.14

Source: PIAAC 2013

^a^ information missing for some country-year combinations

^b^ information not available for France

^c^ information not available for Germany

### 4.1 Distinguishing natives from immigrants

PIAAC asked respondents in what country they were born, and whether their parents had been born outside the country in which they took the PIAAC test. We used this information to distinguish first generation immigrants, 1.5 generation immigrants, and natives. Natives are people whose parents were both born in the country were the respondent lived and was tested. Natives are thus people with no reported cross-national migration history for two generations, plus those born in a foreign country to two natives of the country in which they now live. First generation immigrants are foreign-born respondents with at least one parent born in a country other than the one where they took the PIAAC tests. 1.5 generation immigrants are respondents who were not born in the country where they took the PIAAC test, but who had migrated to that country when they were less than 12 years old [20]. This ensures that the 1.5 generation all had a chance to go through secondary education in the host country. We also include a dummy signifying whether or not people had one parent born in the test country. In the analyses, natives are the omitted reference category. Our analytic sample includes 7,831 first generation and 1,912 1.5 generation immigrants.

### 4.2 Dependent variables: Numeracy and literacy skills

Our dependent variables are derived from the PIAAC measure of competence in numeracy and literacy skills. Numeracy is defined as “the ability to access, use, interpret and communicate mathematical information and ideas in order to engage in and manage the mathematical demands of a range of situations in adult life.” Literacy is “understanding, evaluating, using and engaging with written texts to participate in society, to achieve one’s goals, and to develop one’s knowledge and potential.” The test has 56 numeracy items and 58 literacy items that together measure how well respondents can use mathematical and written information to solve real-life problems [75]. To reduce the total time-on-test, respondents were given only a subset of the items. Item response techniques were then used to compute 10 plausible values for numeracy, and 10 for literacy. These plausible values are psychometric measures of skills. The plausible values allow for direct and unbiased estimation of differences in the numeracy and literacy proficiency of migrants and natives in various countries [31,75,76]. The PIAAC numeracy scale in our data ranges from 24 to 444, with an overall average of 269 and a standard deviation of 50. The literacy scale ranges from 24 to 416, with an average of 272 and a standard deviation of 46.

### 4.3 Independent variables related to composition

We use a number of demographic variables, including a dummy signifying whether a respondent was *living with a spouse or significant other* (1) or not (0). This is the closest we can get to measuring marital status in these data. Missing values (0.03%) were coded as a separate dummy. To measure *health*, the PIAAC survey asks, “In general, would you say your health is excellent, very good, good, fair or poor?” This self-assessment is commonly used in international surveys and is strongly associated with objective indicators of health [77]. We use a dummy distinguishing whether or not respondents were in poor health (1), or in excellent, very good, good, or fair health (0). Missing values (0.1%) are categorized and added as a dummy. *Educational attainment* is the number of years respondents would normally have spent in formal education to obtain the highest credential they have attained. Cross-national comparability is achieved by combining information on respondents’ answer to the question “which qualification on this card is the highest you have obtained” with the reported highest level of education in national education systems. The information was converted into nominal years of schooling by country experts [75]. To measure the *level of education of respondents’ parents*, we use a categorical variable that indicates the level of the most educated parent. We distinguish whether the most educated parent was higher educated (ISCED 5 and 6), medium educated (ISCED 3 and 4), or lower educated (ISCED 1 and 2). Respondents with two lower educated parents form the reference category. A dummy accounts for missing values (5.4%). To identify differences in labor market participation between immigrants and natives, we use a dummy variable coded (1) if respondents are *unemployed* (i.e. not at work but in the labor force and looking for work), and (0) otherwise. Missing values were coded as a separate dummy. *Work experience* is the total number of years in which respondents reported having done paid work during their lifetime. To measure the extent to which people were *speaking a non-native language at home*, respondents were asked about the languages they had learned as a child and still understood. From this information, the OECD determined respondents’ native language. The variable we use is scored (1) if respondents’ native language is different from the language in which the survey was performed,and (0) if it was the same. Missing values (0.2%) were separately coded as a dummy variable.

### 4.4 Independent variables related to birth countries

To account for *origin-related literacy differences* we use the average education levels of all respondents born in that country. For natives, their birth country is by definition the country in which they took the PIAAC test. For first generation immigrants, birth country and destination country differ. Information on birth country literacy is derived from Barro and Lee’s longitudinal data set on educational attainment trends [78]. Their data set (version 2.0) has information on average years of schooling of countries’ populations aged 15 and over. Longitudinal data are available from 1950–2010, but only in five year intervals. We linearly interpolated data to impute the years within these five-year intervals. We used the mean education level of the population in the year natives left the education system as a proxy for individuals’ own education. For migrants, we used the year of migration as reference year.

### 4.5 Independent variables related to destination countries

We measure the extent to which destination countries have adopted *integration policies* to promote the social inclusion of immigrants with the 2010 MIPEX index [79], which measures integration policies in the 17 countries in our data. Based on peer-reviewed information from experts on migration laws, education and discrimination, the index uses information from 148 indicators on laws and policies related to immigrants’ labor market mobility, educational inclusiveness, anti-discrimination regulations, migrants’ political participation, becoming a national, laws regulating family reunion, and long term residence. To measure the extent to which countries’ *education systems* accommodate immigrants’ special needs, we use the percentage of children in schools that have over 25% immigrant children [80]. We also tested alternative measures of countries’ educational system inclusiveness, relying on OECD [80] for information on the number of immigrant students in each country of destination, and on OECD [81]. These analyses led to substantially identical conclusions.

*Labor market protectionism* is measured with the Employment Protection Legislation (EPL) index, developed by the OECD [82]. We use the index scores from 2008 for three indicators: regular contracts, temporary contracts and collective dismissals. The EPL index uses the existence of (a) policies to protect workers against dismissal, (b) requirements for collective dismissals and (c) regulations regarding temporary employment. A higher score corresponds to a more protected labor market. Scores range from 0.85 to 3.11. To estimate the levels of *ethnic* and *cultural diversity*, we use measures devised by Alesina et al [83]. Each measure reflects the probability that two randomly selected people from a destination country are from a different ethnic or cultural group. A higher score indicates a higher level of diversity. The Alesina measures were provided by Teorell et al.[84].

### 4.6 Control variables

We control for gender differences of immigrant and native sub-populations, using a dummy that signifies whether respondents were *male* (1) or female (0).We also control for differences in *age* distributions, and include an additional quadratic term to account for the non-linearity of the relationship between age and skills. The mean age in the entire sample is 41 years. At the macro level, we need to control for contextual factors related to destination country differences in average numeracy. To do so in a parsimonious way, we use the average score on the PIAAC numeracy test.

## 5. Analyses and results

### 5.1 Descriptive analyses: The size of the skills gap

**Fig 1:**
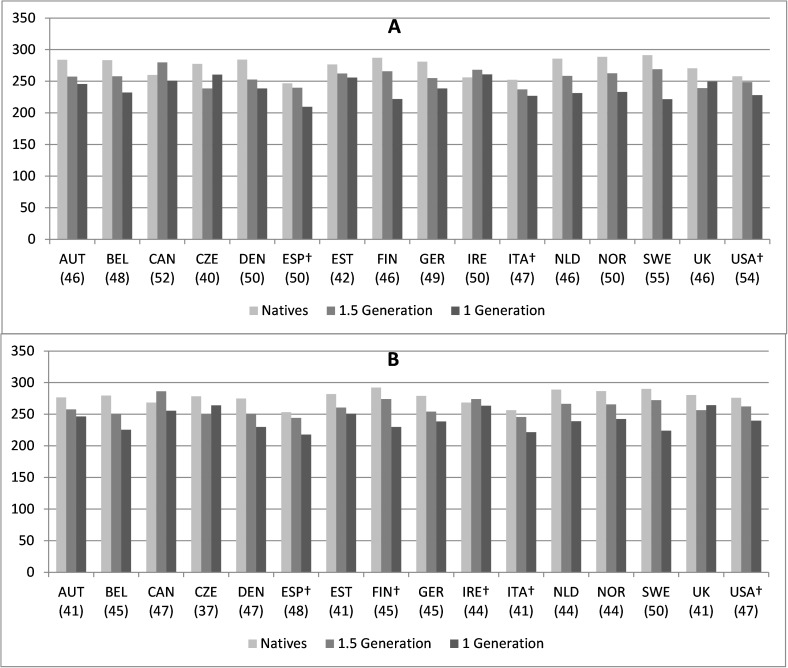
Skills disparities between natives and 1 and 1.5 generation immigrants in 16 OECD countries. A: Numeracy. B Literacy. All differences are statistically significant, unless otherwise stated. † difference between natives and 1.5 generation migrants not statistically significant. Within-country standard deviations in parentheses. Source: PIAAC 2013

Fig 1 shows the numeracy and literacy skill gap between natives, 1.5 generation migrants and first generation migrants in the destination countries we study. The estimates are the result of separate OLS regression analyses for each participating country, controlling whether the migrants had a parent from the destination country. The bars present a graphical depiction of estimated differences in the numeracy and literacy proficiency of migrants and natives in various destination countries. To put the disparities into perspective, recall the overall distribution of numeracy and literacy scores over all respondents in our data. The PIAAC numeracy scale has an overall average of 269 and a standard deviation of 53. The literacy scale has an average of 272 and a standard deviation of 46. Within-country standard deviations are in parentheses.

The upper panel (A) of Fig 1 shows that in most countries, first generation migrants are less proficient in numeracy skills than natives. The gap between natives and first-generation migrants is largest in Sweden, Finland, Norway, Belgium and the Netherlands, with estimated proficiency gaps of about one standard deviation. In Spain, Denmark, Austria and Germany, the gap is about two-thirds of the standard deviation. The smallest numeracy gaps are in Canada and the UK, and in former socialist countries like the Czech Republic and Estonia. Here, gaps amount to about half a standard deviation. In Ireland, first generation migrants are actually slightly more proficient than natives. The difference–although small—is statistically significant. In most countries, 1.5 generation migrants also perform worse than natives, but the gap is usually smaller than the gap between natives and first generation migrants. Notable exceptions are Ireland and Canada, where 1.5 generation immigrants outperform natives b about one-third of a standard deviation. No significant numeracy skills disparities can be observed between 1.5 generation migrants and natives in the USA, Italy and Spain.

The lower panel (B) of Fig 1 shows that cross-national patterns for literacy skill gaps are almost identical to patterns of numeracy skill disparities. We may conclude that numeracy and literacy skills gaps vary almost identically between countries. This may reflect that PIAAC was administered in the official national language(s) of the country (in some cases, in addition to the national language, tests were taken in a widely used other language). Migrants who do not speak that language well would be disadvantaged also in numeracy tests.

Fig 1 also demonstrates that we must take cross-national differences in natives’ proficiency into account in order to explain cross-national variation in the skill gap between natives and immigrants. For example, immigrants to Nordic countries other than Sweden usually have numeracy levels comparable to immigrants in other countries. Nonetheless, there is a large gap between first generation immigrants and natives in these Nordic countries, because natives of these countries have relatively high scores. Conversely, first generation migrants to Italy, Sweden and Spain are the least numerically proficient, but the numeracy gap between first generation immigrants and natives in Italy is relatively small, because Italian natives also have relatively low numeracy skills. However, the numeracy gap between 1.5 generation immigrants and natives is relatively small in Sweden, which may indicate that immigrants who arrive in Sweden as children and attend Swedish schools have a relatively good chance of catching up with native Swedes.

### 5.2 Multilevel analyses

We turn now to exploring whether observed country differences explain cross-national variation in numeracy and literacy gaps and are associated with characteristics of these countries in the way our hypotheses predict. The hierarchical structure of the PIAAC data implies that we can use multilevel models to construct accurate standard errors [32]. We estimate two-level random slope models, with all respondents nested in destination countries. Birth country variables are added at the level of individuals. We experimented with alternative specifications of origin countries, e.g. by adding origin dummies. This method provides a more conservative control for unobserved heterogeneity driven by origin group differences, but precludes testing substantive hypotheses on the relevance of country-of-origin differences. In these models, the cross-national variation of numeracy skills depicted in Fig 1 is accounted for by a variance component that estimates the variance at the level of countries. In addition to the random intercept, we allow the estimated differences between natives and first or 1.5 generation migrants to vary between countries by modelling random slopes for these parameters. We used the appropriate methods for analyzing plausible values. To do so, we analyzed the data with the statistical software package HLM2 [85].

Table 2 presents our results for numeracy skills. Table 3 presents these results for literacy. Model 1a estimates numeracy skill disparities between natives and first and 1.5 generation immigrants in a cross-national design. In Model 1b, we present similar analyses for reading literacy. Overall, first generation immigrants have significantly lower numeracy skills than natives (Model 1a: *b* = -35.425). Generation 1.5 immigrants are also less proficient than natives, but the gap is only half as large (*b* = -17.232). The significance of the random components indicates that the skill disparities between natives and both first generation and 1.5 generation immigrants differ systematically between countries (*Ω^2^1g* = 412 and *Ω^2^1*.*5g* = 201)). The random intercept makes clear that, as is usual in these analyses, most of the variance is between individuals within countries (*Ω^2^i* = 2488). Only about 8% of the variance is generated at the country level (*Ω^2^c* = 208). Model1b in Table 3 shows that results for literacy are highly similar.

**Table 2:** Multilevel regression of explanatory variables on numeracy.

	Model 1a		Model 2a		Model 3a		Model 4a		Model 5a^≠^		Model 6a		Model 7a		Model 8a^†^	
Intercept	273.365	***	187.253	***	50.080		36.220		51.224		19.426		63.648		58.245	
**Skills gap**																
Non-immigrants (ref.)																
1 generation immigrant	- 35.425	***	-24.558	***	-42.936	***	-16.824		-10.593	*	9.858		-15.818	***	-17.414	***
1.5 generation immigrant	-17.232	**	-7.443	*	-8.401		14.725		-23.537	**	-2.668		-41.024	***	-38.249	***
**Individual-level variables**																
One native parent	9.999	***	8.567	***	7.872	**	7.855	*	8.045	**	7.814	**	10.380	***	10.394	***
Male			11.508	***	11.709	***	11.676	***	11.834	***	11.675	***	11.658	***	11.621	***
Age			-0.407	***	-0.339		-0.269		-0.192		-0.269		-0.273		-0.258	
Age^2^			-0.005	***	-0.005		-0.005		-0.005	*	-0.005		-0.005	*	-0.005	
Living together/married(single = ref)			5.582	***	5.528	***	5.595	***	5.467	**	5.593	***	5.543	***	5.608	***
Poor health (better health = ref.)			-14.591	***	-14.746	***	-14.823	***	-15.139	***	-14.815	***	-14.878	***	-14.482	***
Educational attainment			7.015	***	6.904	***	6.804	***	6.705	***	6.803	***	6.807	***	6.800	***
Parents lower educated (ref.)																
Parents middle education			7.295	***	7.020	***	7.136	***	7.114	***	7.144	***	7.287	***	7.563	***
Parents higher education			17.986	***	17.722	***	17.883	***	17.955	***	17.893	***	18.016	***	18.106	***
Parents education unknown			-8.293	***	-8.511	***	-8.453	***	-8.967	***	-8.440	***	-8.398	***	-7.977	***
Unemployed (employed = ref.)			-8.618	***	-8.625	***	-8.715	***	-9.056	***	-8.716	***	-8.745	***	-8.326	***
Work experience			0.440	***	0.415	***	0.425	***	0.428	***	0.425	***	0.434	***	0.447	***
Foreign language spoken at home			-14.053	***	-13.491	***	-13.436	***	-12.880	***	-13.470	***	^a^		^a^	
**Origin countries**																
General education level					0.484		1.437	**	1.640	**	1.445	**	1.528	**	1.327	**
General education leve*1 gen					0.480											
General education level*1.5 gen					2.359	**										
**Destination countries**																
Average numeracy score					0.490	***	0.476	**	0.450	**	0.489	**	0.409	**	0.427	**
Integration policies							0.110									
Integration policies*1 gen							-0.623	*								
Integration policies *1.5 gen							0.254									
Inclusive education system									-0.287							
Inclusive education system *1 gen									0.154							
Inclusive education system *1.5 gen									0.590	***						
Protected labor market											9.183	**				
Protected labor market*1 gen											-9.098	**				
Protected labor market *1.5 gen											-5.586					
Ethnic diversity													-17.605			
Ethnic diversity*1 gen													38.101	**		
Ethnic diversity*1.5 gen													29.301	**		
Cultural diversity															-8.416	
Cultural diversity*1 gen															28.713	
Cultural diversity*1.5 gen															35.914	*
**Random effects**	Ω		Ω		Ω		Ω		Ω		Ω		Ω		Ω	
Individuals [Ω]	2488	***	1760	***	1748	***	1750	***	1743	***	1750	***	1754	***	1757	***
Countries [Ω]	208	***	172	***	98	***	119	***	116	***	77	***	117	***	129	**
1 generation slope [Ω^2^_1g_]	412	***	205	***	180	***	162	***	207	**	173	***	180	***	234	**
1.5 generation slope [Ω^2^_15g_]	201	***	71	***	63	***	63	***	24	***	58	***	31	***	46	*

*Notes*: Estimates are multilevel regression coefficients on plausible values of numeracy scores.

*** p < 0.0001

** p<0.001

* p <0.05

^a,^not included in model for reasons of overcontrolling

^≠^ Model does not include data from France

^†^ Model does not include data from Germany. Source: PIAAC 2013

**Table 3:** Multilevel regression of explanatory variables on literacy.

	Model 1b		Model 2b		Model 3b		Model 4b		Model 5b^≠^		Model 6b		Model 7b		Model 8b^†^	
Intercept	276.229	***	211.486	***	86.183	*	75.865		68.890		81.113	*	88.738	*	69.907	
**Skills gap**																
Non-immigrants (ref.)																
1 generation immigrant	-33.550	***	-24.851	***	-41.603	***	13.555		-24.709	***	-6.746		-37.983	***	-35.352	***
1.5 generation immigrant	-15.036	***	-8.666	**	-6.671		-11.729		-9.307	**	11.086	*	-13.028	***	-12.177	**
**Individual-level variables**																
One native parent	11.898	***	9.675	***	9.492	***	9.444	***	9.796	***	9.285	***	11.948	***	11.960	***
Male			1.546	***	1.828	***	1.798	***	2.034	***	1.797	***	1.781	***	1.789	***
Age			-0.330	***	-0.284	***	-0.224	**	-0.177	*	-0.221	**	-0.225	**	-0.201	**
Age^2^			-0.004	***	-0.004	***	-0.004	***	-0.005	***	-0.004	***	-0.004	***	-0.005	***
Living together/married(single = ref)			3.280	***	3.124	***	3.184	***	3.206	***	3.181	***	3.129	***	3.108	***
Poor health (better health = ref.)			-14.095	***	-13.572	***	-13.644	***	-13.706	***	-13.633	***	-13.700	***	-13.458	***
Educational attainment			5.895	***	5.921	***	5.837	***	5.817	***	5.828	***	5.836	***	5.836	***
Parents lower educated (ref.)																
Parents middle education			7.152	***	7.420	***	7.526	***	7.384	***	7.530	***	7.667	***	7.995	***
Parents higher education			17.357	***	17.542	***	17.685	***	17.660	***	17.692	***	17.810	***	17.898	***
Parents education unknown			-6.175	***	-6.035	***	-5.979	***	-6.729	***	-5.973	***	-5.934	***	-5.317	***
Unemployed (employed = ref.)			-5.327	***	-5.604	***	-5.686	***	-5.772	***	-5.686	***	-5.715	***	-5.454	***
Work experience			0.199	***	0.217	***	0.226	***	0.235	***	0.227	***	0.235	***	0.247	***
Foreign language spoken at home			-14.091	***	-13.453	***	-13.394	***	-12.824	***	-13.450	***				
**Origin countries**																
General education level					0.868	***	1.679	***	1.931	***	1.727	***	1.788	***	1.577	***
General education level*1 gen					0.327											
General education level*1.5 gen					2.091	***										
**Destination countries**																
Average numeracy score					0.426	**	0.385	*	0.446	**	0.379	*	0.382	*	0.451	**
Integration policies							0.202									
Integration policies*1 gen							-0.619	**								
Integration policies *1.5 gen							0.169									
Inclusive education system									-0.100							
Inclusive education system *1 gen									0.117							
Inclusive education system *1.5 gen									0.518	**						
Protected labor market											3.650					
Protected labor market*1 gen											-7.771	*				
Protected labor market *1.5 gen											-6.111	*				
Ethnic diversity													-7.302			
Ethnic diversity*1 gen													22.033			
Ethnic diversity*1.5 gen													19.761	*		
Cultural diversity															2.946	
Cultural diversity*1 gen															12.243	
Cultural diversity*1.5 gen															16.234	
**Random effects**																
Individuals [Ω]	2057.789	***	1507.804	***	1490.468	***	1491.838	***	1491.482	***	1493.476	***	1496.981	***	1498.961	***
Countries [Ω]	105.862	**	72.011	**	49.949	**	52.173	**	52.738	**	51.953	**	56.310	**	52.956	**
1 generation slope [Ω^2^_1g_]	288.494	**	119.569	**	100.623	**	69.406	**	121.004	**	85.647	**	121.046	**	137.406	**
1.5 generation slope [Ω^2^_15g_]	112.780	*	57.377	*	38.399	*	37.132	*	10.831	*	25.916	*	23.015	*	34.241	*

*Notes*: Estimates are multilevel regression coefficients on plausible values of literacy scores.

*** p < 0.0001

** p<0.001

* p <0.05

^a,^not included in model for reasons of overcontrolling

^≠^ Model does not include data from France

^†^ Model does not include data from Germany. Source: PIAAC 2013

In Models 2a and 2b, we add demographic and socioeconomic variables and control variables. The parameters behave as expected. Model 2a shows that numeric proficiency initially rises with age, peaks at about age (-0.407/(-0.005*2) = 40,7 and then declines. Men are also more proficient in numeracy then women (*b* = 11.508), and those living with a spouse or partner are more proficient than those living alone (*b* = 5.658). People in poor health are much less numerically proficient than those with fair health or better (*b* = -14.591). Educational attainment is positively related to skills (*b* = 7.015). Over and above respondents’ own education, parental education is also positively related to numeracy. In addition, the unemployed are less numerically skilled (*b* = -8.618), and people with longer work experience are more skilled (*b* = 0.440). Finally, those whose native language is not that of the destination country perform worse on the numeracy tests (*b* = -14.053). In Model 2b we observe similar coefficients for reading literacy.

So how does controlling for these characteristics contribute to explaining the overall gap between first and 1.5 generation migrants and natives? We focus on numeracy to show how this works. The estimates of random components in Model 2a are somewhat lower than those in Model 1a. Compared to Model 1a, the cross-country variance of the slope of being a first generation immigrant is reduced considerably, from *Ω1g* = 412 to *Ω 1g* = 205. Variables related to demographics, educational attainment, parental background and employment account for over half of the total cross-national variance in the size of the gap between first generation immigrants and natives and about two thirds of the variance of the random slope of 1.5 generations. Comparing variance components across models must be done with caution, especially when dealing with small sample sizes. Nonetheless, these observations do not contradict Hypotheses 1a or 1b.

After controlling for these background characteristics, the cross-national variation of the skills gaps are not fully explained. This leaves room for the contextual explanations we argued could be relevant. We now turn to testing hypotheses on country characteristics on the skills gap. To do so, we start with the specification of Models 2a and 2b and, in subsequent steps, separately add country level variables and their interactions with first and 1.5 generation immigrants. The extent to which the immigrant gradients covary with the country-level characteristics is informative about the plausibility of our hypotheses. In general, we may interpret significant fixed interactions as supporting evidence of the relevance of the macro-level indicator for explaining skills gaps. The random slopes are informative about the extent to which the interaction terms in the models help explain cross-national differences in the skill disparities between natives and immigrants. All the models control for compositional differences related to demographics, educational attainment, and socioeconomic background. We also control for countries’ average score on the PIAAC numeracy index, to control parsimoniously for unobserved country-level differences that affect mean numerical literacy.

In Models 3a and 3b, we add variables related to the average educational attainment of the countries where natives and immigrants were born. Being born in countries with higher education levels is positively related to ones’ numeracy (Model 3a: *b* = 0.484) and literacy (Model 3b: *b* = 0.868) proficiency. The parameter predicting numeracy skills is insignificant. The skills gap between first generation migrants and natives is also not significantly associated with differences in the educational level of natives in origin countries, both for numeracy and literacy. For the 1.5 generation, bot numeracy and literacy skill gaps are smaller in countries with more immigrants from countries in which the mean education level of the population resembles that of the destination country. Origin countries’ average education level also contributes to interpreting the cross-national variance of the skills gaps. In general, our findings are in line with Hypotheses 2a and 2b and support the assumption that origin country differences remain important for predicting how first generation immigrants fare in destination countries relative to natives, but the insignificance of the estimates for explaining skills gaps between first generation migrants and natives indicates that the evidence is weak. For migrants who migrated before age 12, the data are somewhat more convincing.

Model 4a shows that the numeracy gap between migrants and natives varies systematically with the extent to which countries have adopted societal integration policies for immigrants. After controlling for a vast number of individual-level predictors of numeracy scores, we observe a negative cross-level interaction effect for first generation immigrants. This indicates that first generation migrants fare worse compared to natives in countries that have stronger integration policies (*b* = -0.623). Model 4b in Table 3 show similar parameters for literacy gaps. These findings may demonstrate that countries adopt integration policies in reaction to stagnating integration, and that these policies have not (or not yet) had the desired effect. The interactions are insignificant for 1.5 generation immigrants.

In Models 5a and 5b, we focus on educational systems. We hypothesized that numeracy and literacy skills disparities between natives and 1.5 generation immigrants would be smaller in countries that are better equipped to accommodate the educational needs of immigrant children. In Models 5a and 5b, this appears to be the case (*b* = 0.590 for numeracy, *b* = 0.518 for literacy). We conclude that hypotheses 4a and 4b are supported.

We also expected that the numeracy and literacy skill gaps between natives and 1.5 generation immigrants would be larger in countries with stronger labor market protection for workers (5). The estimates in Models 6a and 6b are in line with this reasoning. The negative interactions for first generation immigrants (*b* = -9.096) in Model 6a suggests that the numeracy gap between them and natives is indeed wider in countries with more protective labor markets. For the 1.5 generation, the point estimate is also in the predicted negative direction, but the coefficient is not significant (*p* = 0.160). In Model 6b, we observe similarly negative interactions for literacy, now both significant. The results in these models suggest that immigrants who arrive in destination countries before the age of 12 have better access to the labor markets of destination countries than those who arrive during or after adolescence.

Moving on to the role of destination countries’ ethnic and cultural context, hypotheses 6a and 6b used ethnic constrict theory to predict that numeracy and literacy disparities between natives and first and 1.5 generation immigrants would be larger in countries with more ethnic diversity. Hypotheses 7a and 7b, derived from ethnic contact theory, predicted the opposite. Model 7a provides evidence that the numeracy gap between first (*b* = 38.101) and 1.5 (*b* = 29.301) generation immigrants and comparable natives is smaller in more ethnically diverse countries. Model 7b shows that the literacy gap between first (*b* = 22.033) and 1.5 (*b* = 19.716) generation immigrants and comparable natives is smaller in more ethnically diverse countries. Estimates are in the expected direction, although not or not highly significant. Hypotheses 7a and 7b find support, and hypotheses 6a and 6b are rejected. However, given the low significance of estimates for literacy gaps, we would argue that evidence is rather weak.

Hypotheses 8a and 8b predicted that multiculturalism would hamper the assimilation of immigrants. The coefficients in Model 8a show that the numeracy gap between 1.5 generation immigrants and natives is slightly smaller in more culturally diverse countries (*b* = 35.914), which is inconsistent with the hypothesis’ claim that multiculturalism hampers assimilation of immigrants. The interaction for the first generation is not significant. Model 8b in Table 3 shows no significant relation between the level of cultural diversity and literacy differences between immigrants and natives. However, accounting for cultural diversity does help to explain part of the cross-national difference in numeracy and literacy gaps between first generation migrants and natives.

## 6. Conclusions

This paper has tested various theoretical explanations for numeracy and literacy skill disparities between adult immigrants and natives using cross-national data on numeracy and literacy skills from 85,875 adults aged 16–65 in 17 countries. We have shown that adult immigrants are less skilled than non-immigrants in all but one of the 17 Western countries we examined. The skill gaps between natives and first-generation migrants are largest in the Nordic countries, and the Netherlands, with estimated proficiency gaps of over one standard deviation. The gaps are smallest in Canada, the UK and formerly socialist countries in Eastern Europe. First generation migrants are more numerically proficient than natives in Ireland.

Adult 1.5 generation immigrants also perform worse than natives on numeracy and literacy, but the gap is smaller than the gap between natives and first generation migrants who arrived as adults, and in some cases it is reversed. No significant numeracy and literacy skills disparities can be observed between natives and 1.5 generation migrants in Italy, Spain or the United States. In Canada and Ireland, 1.5 generation immigrants outperform natives.

What do these gaps mean? The PIAAC was administered in national languages of the test countries (or other dominant languages), so performance gaps between natives and immigrants are not surprising. The OECD [8] notes that “relatively low scores of immigrants in the test language(s) among non-native speakers of those languages, such as immigrants and their children, is [sic] not necessarily indicative of poor performance”. However, that does not mean that gaps are not important. To participate economically and socially in destination countries, immigrants (usually) have to read and work with numbers in the language(s) that are most relevant to these countries. Proficiency gaps in numeracy and literacy may indicate that immigrants are probably less able to put their skills to productive use.

We analyzed observed cross-national differences with multilevel analyses. This strategy helps to empirically assess the extent to which various theories contribute to explaining cross-national differences in skill disparities. For all the benefits multilevel analyses have over non-hierarchical regression models, one important caveat about our results requires emphasis. Multilevel models assume exogeneity of parameters rather than econometrically excluding the possibility that parameters are correlated with error terms. That means that our findings should not be treated as definitive evidence about the impact of particular policy changes in particular countries. We cannot answer such causal questions with this data. To address specific follow-up research questions regarding the size of causal effects, future research could rely on natural experiments within countries and quasi-experimental methods that can better exclude endogeneity. For example, an instrumental variables approach exploiting exogenous contextual variation within countries over time may provide better causal estimates. In the meantime, our analyses offer quantitative analyses that are more informative than those previously available about the likelihood that the theories we have considered can explain the observed variation in skill gaps between immigrants and natives in Western countries.

Our analyses are consistent with three general theoretical conclusions. First, compositional differences partly explain the numeracy and literacy gaps between immigrants and natives in rich Western countries. Population differences regarding demographic makeup, migrants’ and natives’ educational attainment, their parental background, and their employment account for about half the total cross-national variation of skills disparities between first generation immigrants and natives, and about two thirds of the variation in disparities between 1.5 generations and natives. However, these background characteristics do not fully explain cross-national variation in these gaps. This implies that contextual differences are also important for understanding skill gaps. This argument has also been made by others [45], but we are the first to demonstrate this cross-nationally on large samples of first generation migrants and natives.

Our second theoretical conclusion is that receiving countries’ ethnic diversity matters. We find smaller gaps in more ethnically diverse countries. Although we could not test this directly, we think it is implausible that this regularity can be explained by selective migration: it is not clear why migrants who are more like natives in terms of skills would migrate to more ethnically diverse countries. Our observations seem to be more informative about social-scientific theories that point toward the relevance of ethnic diversity for the integration of immigrants after migration. Theories about the consequences of mass migration for receiving countries sometimes predict that greater ethnic or cultural diversity in destination countries will have negative externalities. That may be true in some domains, but our analyses suggest that ethnic diversity predicts narrower numeracy skills gaps between natives and immigrants. Our estimates for reading literacy are less pronounced, but point in the same direction. This implies that hypotheses derived from ethnic constrict theory [36] find no support by our analyses. Our analyses also do not support theoretical notions, advanced by Koopmans [66](2013) for example, that multiculturalism hampers immigrant integration. This may be explained by the fact that in more culturally diverse societies, the boundaries between natives and minority cultural groups become more blurred, facilitating immigrant assimilation [86]. This interpretation is also consistent with our finding smaller skills gaps in more ethnically diverse countries.

Our third theoretical conclusion is that the size of skill disparities between immigrants and natives depends partly on institutional differences between receiving countries. Skills gaps are smaller in countries where labor markets are less protected, and where education is better suited for educating immigrants. Of all the tested variables, the insider-outsider distinction that accompanies labor market protection provides the best explanation for cross-national variation in skills gaps between first generation migrants and natives. For 1.5 generation migrants, the extent to which the educational system is accustomed to dealing with migrants is key. We measured this as the percentage of students in schools with high concentrations of immigrants). In additional analyses we experimented with different measurements, such as the math gap between immigrants and native 15-year olds, and the percentage of schools with large proportions of immigrants. Our conclusions are insensitive to measurement of this variable.

These conclusions, while tentative, have two potential implications for policies aimed at reducing inequalities between migrants and non-migrants. First, in the long run, selective immigration policies may go a long way in reducing skills gaps. Point systems and human capital related immigration policies remain contested policy territory. We did not directly test hypotheses about point systems, but our results are certainly compatible with the notion that selecting migrants based on background characteristics such as their educational attainment and likely employability may reduce aggregate skill inequalities in the longer run.

However, these policies will do little to affect inequality between natives and migrants who are already part of the receiving society. Here, other contextual characteristics come into play. Skills disparities appear to be smaller in countries that are better equipped to deal with ethnic diversity. However, these societal properties are not easily affected by policy. Our analyses do not suggest that policies specifically aimed at encouraging immigrant integration have had their desired effect. We found that skills gaps, if not unaffected, are larger in countries that have more elaborate integration policies. We doubt that such policies actually have the opposite effect from what they intend. However, it is plausible that countries adopt stronger integration policies when they have more severe integration problems with immigrants. If that is the case, these policies may make a modest contribution to integrating immigrants, but their effect may not be large enough to solve the problems they were meant to solve.

For the 1.5 generation, education seems paramount. Our analyses clearly show that the gaps are much smaller in countries where a larger part of the student population is in schools with high concentrations of immigrants. This finding suggests that inequalities between adult migrants and natives are smaller in countries where the educational system is more accustomed to dealing with the particular challenges of educating immigrant children. It might of course also be the case that in such systems, native children become more like immigrants. However, additional analyses on a subsample of native children reveal a positive relation between the average percentage of immigrant children in schools and native children’s performance on numeracy perform slightly better in countries where a higher percentage of the student population is immigrant.

Furthermore, skills gaps between first generation migrants and natives are smaller in countries with less protected labor markets. Future studies testing the causal impact of educational and labor market characteristics on skill disparities between immigrants and natives should establish whether institutional reforms in these fields can actually serve to narrow skills gaps between migrants and natives.
